# Risk of Birth Defects in Australian Communities with High Levels of Brominated Disinfection By-products

**DOI:** 10.1289/ehp.10980

**Published:** 2008-04-18

**Authors:** Kimberley Chisholm, Angus Cook, Carol Bower, Philip Weinstein

**Affiliations:** 1 Environmental Determinants of Chronic Disease Group, School of Population Health; 2 Telethon Institute for Child Health Research, Centre for Child Health Research, The University of Western Australia, Perth, Australia; 3 Western Australian Birth Defects Registry, Women and Infant Health Service, Perth, Australia

**Keywords:** birth defects, disinfection by-products, epidemiology, pregnancy, trihalomethanes (THMs)

## Abstract

**Background:**

By international standards, water supplies in Perth, Western Australia, contain high trihalomethane (THM) levels, particularly the brominated forms. Geographic variability in these levels provided an opportunity to examine cross-city spatial relationships between THM exposure and rates of birth defects (BDs).

**Objectives:**

Our goal was to examine BD rates by exposure to THMs with a highly brominated fraction in metropolitan locations in Perth, Western Australia.

**Methods:**

We collected water samples from 47 separate locations and analyzed them for total and individual THM concentrations (micrograms per liter), including separation into brominated forms. We classified collection areas by total THM (TTHM) concentration: low (< 60 μg/L), medium (> 60 to < 130 μg/L), and high (≥ 130 μg/L). We also obtained deidentified registry-based data on total births and BDs (2000–2004 inclusive) from post codes corresponding to water sample collection sites and used binomial logistic regression to compare the frequency of BDs aggregately and separately for the TTHM exposure groups, adjusting for maternal age and socioeconomic status.

**Results:**

Total THMs ranged from 36 to 190 μg/L. A high proportion of the THMs were brominated (on average, 92%). Women living in high-TTHM areas showed an increased risk of any BD [odds ratio (OR) = 1.22; 95% confidence interval (CI), 1.01–1.48] and for the major category of any cardiovascular BD (OR = 1.62; 95% CI, 1.04–2.51), compared with women living in low-TTHM areas.

**Conclusions:**

Brominated forms constituted the significant fraction of THMs in all areas. Small but statistically significant increases in risks of BDs were associated with residence in areas with high THMs.

Disinfection by-products (DBPs) are a major group of water contaminants, and their role in causing adverse health outcomes has been subject to extensive epidemiologic and toxicologic research and review (Bove et al. 2002; Butterworth 2005; Butterworth and Bogdanffy 1999; Goebell et al. 2004; Graves et al. 2001; Nieuwenhuijsen et al. 2000; Reif et al. 1996; Tardiff et al. 2006; Villanueva et al. 2003). Determination of safe levels in drinking water has been subject to extensive debate, with a wide range of acceptable levels set across the industrialized world [Keegan et al. 2001; National Health and Medical Research Council (NHMRC) 2004; Rodriguez et al. 2004; U.S. Environmental Protection Agency (EPA) 2004].

DBPs are formed by the reaction of chemical disinfectants with natural organic matter (NOM) in water (Piccolo and Spiteller 2003; Rook 1974; Thurman and Malcolm 1981). The structure and quantity of NOM in water sources—and hence the quantity and type of DBPs formed—vary with geographic location and season (Chen and Weisel 1998; Rodriguez and Serodes 2001). DBPs commonly found in drinking water include trihalomethanes (THMs) [often measured as total trihalomethanes (TTHMs)], halogenated acetic acids (HAAs), and halogenated acetonitriles (HANs), although TTHMs have most often been the focus of previous toxicologic and epidemiologic research. Research to date has focused on the four regulated THMs: chloroform, bromoform, bromodichloromethane (BDCM), and dibromochloromethane (DBCM) (Da Silva et al. 2000). Toxicologic studies have shown THMs may result in adverse effects in laboratory animals, including hepatotoxicity, nephrotoxicity, mutagenesis, carcinogenicity, and adverse reproductive effects (DeAngelo et al. 2002; Geter et al. 2004; International Life Science Institute 1995; Keegan et al. 1998; Landi et al. 1999; Agency for Toxic Substances and Disease Registry 1993).

Maternal DBP exposure has been linked to an elevated risk for a range of reproductive outcomes, including pregnancy loss, prematurity, and low birth weight (Dodds et al. 1999, 2004; Kallen and Robert 2000; King et al. 2000, 2005; Waller et al. 1998; Yang et al. 2000), as well as neural tube defects (Dodds et al. 1999; Dodds and King 2001; Hwang and Jaakkola 2003; Klotz and Pyrch 1999), cardiac defects (Hwang et al. 2002), urogenital defects (Magnus et al. 1999), and oral cleft defects (Dodds et al. 1999). Table 1 summarizes the most recent research on DBP exposure and birth defects (BDs). Importantly, these studies show significant effects for several adverse birth outcomes at levels of exposure to THMs that have been observed in Australian metropolitan areas, such as Perth (Dodds et al. 1999; King et al. 2000).

Overall, findings relating to adverse effects on reproductive health have been equivocal and are often limited by crude exposure measures or implausible retrospective estimates of exposure to DBPs. Because concentrations of NOM and other chemical precursors in source waters vary in both time and place, DBP levels also fluctuate, complicating exposure assessment in epidemiologic studies. Several authors have indicated a number of concerns with existing studies, particularly regarding their failure to account for all exposure pathways or the temporal and spatial variability in DBPs within regions of water supply (Graves et al. 2001; Nieuwenhuijsen et al. 2000; Reif et al. 1996; Shaw et al. 2003). Also, in general, studies have not allowed for residential mobility, use of alternative water sources for drinking and bathing, or exposure to water sources outside the home. Dermal absorption of THMs is particularly poorly estimated despite evidence indicating that showering and other forms of immersion are significant sources of exposure (Nieuwenhuijsen et al. 2002; Weisel and Jo 1996; Xu et al. 2002).

Given the ongoing controversy surrounding DBPs and adverse pregnancy outcomes, especially regarding associated BDs, a precautionary approach to DBP exposure during pregnancy is somewhat justified. Most industrialized countries have regulated the permitted levels of TTHM in drinking water, and many of these limits are well below the 250 μg/L level currently accepted in Australia (NHMRC 2004): Canada, 80 μg/L (Rodriguez et al. 2004); United States, 80 μg/L (U.S. EPA 2004); and the United Kingdom, 100 μg/L (Keegan et al. 2001). By international standards, a number of Australian water supplies continue to contain elevated DBP levels. The water supplied to Perth, Western Australia, is distinctive in several respects (Heitz et al. 2001). Depending on the suburb, the residential supply may be derived from groundwater from aquifers underlying the city, surface water from catchments along the eastern edge of the city, or a mixture of both. Shallow groundwater resources in Perth contain both high levels of the dissolved fraction of NOM [dissolved organic carbon (DOC), 10–50 mg/L] and highly variable concentrations of bromide (0–2.2 mg/L), leading to a high fraction of brominated DBP formation (Heitz et al. 2001). As a percentage of NOM, the DOC in groundwater can range from 7% to 77% (Wong et al. 2002). Historical records from the city’s water authority, the Water Corporation of Western Australia, indicate that THM levels range from > 200 μg/L in some water distribution areas to < 4 μg/L in others. The geographic variation in these levels provides an opportunity to examine the relationship between DBP exposure and adverse outcomes of pregnancy within a discrete geographic area. In this study we compared the prevalence of BDs in urban populations whose residential water supplies contained markedly different THM levels.

## Materials and Methods

### Study design

The study used a record-based prevalence design based on the Western Australia Midwives’ Notification System and Birth Defects Registry for the years 2000–2004 inclusive.

### Water sampling and analysis

**Figure 1:**
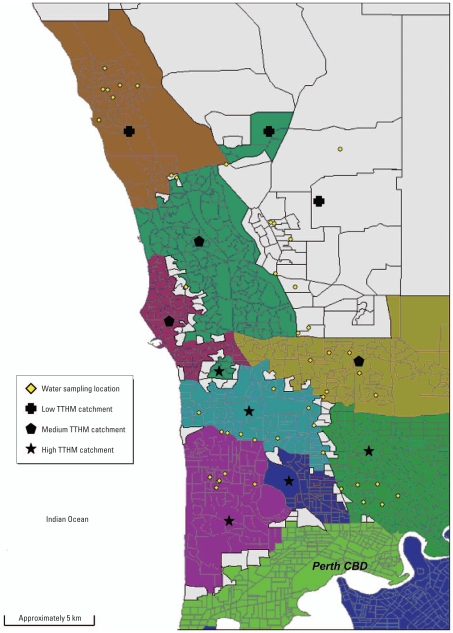
Map of the northern metropolitan area of Perth outlining individual sampling sites. Shaded areas indicate water catchments.

We collected water samples at 47 separate locations within the greater Perth metropolitan area in Western Australia (Figure 1) from publicly accessible taps in highly populated areas, including drinking fountains, outdoor taps close to picnic areas in public parks, and cold-water taps located in public toilets, public parks, and popular restaurants. We chose sampling sites at locations where taps would be frequently used, thereby limiting the storage time of water in delivery pipe networks.

We collected the water samples on six separate occasions from April 2005 to March 2006, inclusive. We flushed taps at each location for 2 min before collection. We collected water samples in 40-mL glass vials, each dosed with the quenching agent sodium thiosulfate, to prevent further THM formation once we took samples. We stored the collected samples on ice in an enclosed insulated carrier. During each collection, we filled vials to maximum level to prevent air bubbles upon sealing, to prevent loss of THMs due to volatilization. We collected samples from all 47 locations within an 8-hr period on each sample day and delivered them to the Chemistry Centre of Western Australia for analysis at the completion of each sampling run.

We performed THM water analyses using the “purge and trap” technique for sample concentration and delivery, followed by gas chromatography with mass spectrometric detection (GC/MS) for the separation and quantification of each of the four analytes (U.S. EPA 2003). In this system, the sample is purged with an unreactive gas and the analytes trapped at a low temperature on an adsorbent trap. The trap is then heated rapidly and the desorbed compounds refocused at the head of a GC chromatographic column for GC/MS analysis. An internal standard is used to correct for slight differences in temperatures and trapping conditions throughout the run. The method is based on the U.S. EPA 5030C (purge-and-trap for aqueous samples) method (U.S. EPA 2003) and is accredited by the Australian National Association of Testing Authorities. Routine quality controls are performed with each batch of samples, such as replicate analyses, sample spiking, and blank analyses. We analyzed each sample for TTHM concentration (in micrograms per liter) and for the four individual THMs chloroform, bromoform, DBCM, and BDCM.

### Data on births and BDs

We obtained number of total births and BDs from post codes corresponding to the water sample collection sites for the years 2000–2004 inclusive from the Western Australia Midwives’ Notification System and Birth Defects Registry, respectively (Bower et al. 2007). These two data sets form part of the comprehensive Western Australia Linked Database, which systematically links available administrative health data within the state using probabilistic matching of patient names and other identifiers (Holman et al. 1999). We obtained deidentified records on birth date, post code of maternal residence at time of birth, maternal age, socioeconomic status code for the maternal residence at time of birth (from linked census data), and the presence of BDs. The Western Australia Birth Defects Registry uses multiple sources of case ascertainment to identify BDs in live births, stillbirths, and pregnancies terminated with fetal abnormality. We did not include minor malformations in our analyses unless they were disfiguring or required treatment. Cases with only minor malformations account for approximately 10% of all BDs registered. We did not include terminations in the present data, but these comprise a relatively small proportion of all cases and have been relatively constant for the past few years.

The Midwives’ Notification System is statutory and is completed for all live births and stillbirths of ≥ 20 weeks’ gestation or birth weight ≥ 400 g. The Birth Defects Registry in Western Australia has a high level of case ascertainment (Bower et al. 2000, 2001).

A BD is defined by the registry as a structural or functional abnormality that is present at conception or occurs before the end of pregnancy and is diagnosed by 6 years of age. We coded each recorded BD using the British Paediatric Association (BPA) International Classification of Diseases, version 9, system (British Paediatric Association 1979). We investigated associations of DBPs with any BD and also with the following major groups of BDs: nervous system defects (BPA codes 74000–74299), cardiovascular defects (BPA 74500–74799), respiratory system defects (BPA 74800–74899), gastrointestinal defects (BPA 74900–75199), urogenital defects (BPA 75200–75399), musculoskeletal defects (BPA 75400–75699), and congenital anomalies of integument (BPA 75700–75799). Because of low case frequencies, we did not examine the following groups separately: congenital anomalies of eye (BPA 74300–74399); congenital anomalies of ear, face, and neck (BPA 74400–74499); chromosome defects (BPA 75800–75899); and all other defects (BPA 24390–24399, 25520–25529, 27000–27099, 27010, 27100–27199, 27700, 28220, 28240–28249, 28600–28620, 35900–35999, 75992, 77100, 77800).

### Data analysis

We used STATA version 9.0 (StataCorp, College Station, TX, USA) for data analysis. We classified collection sites and post codes of maternal residence in three exposure areas: low (TTHM < 60 μg/L), medium (TTHM > 60 to < 130 μg/L), and high (TTHM ≥ 130 μg/L). We the calculated prevalence proportions for any BD and categories of major BDs for low, medium, and high exposure groups. We calculated odds ratios (ORs) and 95% confidence intervals (CIs) for any BD and for categories of BDs using binomial logistic regression models, and calculated estimated risks for the medium and high exposure areas using the low exposure area as the comparison group. We adjusted all estimates for maternal age at an individual level using the deidentified and highly complete case data from registry and midwives’ records and data on socioeconomic status [using Socio-Economic Indexes for Areas (SEIFA) coding (Australian Bureau of Statistics 2001)]. SEIFA values are supplied by post code and are calculated based on variables including the proportion of families with high incomes, people with a tertiary education, and employees in skilled occupations; low values indicate areas of disadvantage, and high values indicate areas of advantage.

## Results

### TTHM levels

We averaged measurements for each collection site over the six collection dates, and identified all post codes as low, medium, or high exposure as described in “Materials and Methods.” Average TTHM concentrations (±1 SD) for low, medium, and high exposure areas were 54 ± 16.6 μg/L, 109.3 ± 28.9 μg/L, and 137 ± 24 μg/L, respectively. Table 2 summarizes area THM levels, including individual THM concentrations. We also assessed the THM levels within each assigned exposure category using analysis of variance. The mean square errors and *F*-test value (*F* = 204292.41; *p* < 0.00001) indicated highly significant between-group differences and relatively little within-group variability for the specified category cutoff points. Preliminary analysis of HAAs and HANs during the December testing month showed very low HAA and HAN concentrations by international standards (data not shown), so we did not continue monitoring these compounds.

### Prevalence of BDs

A total of 1,097 individuals with BDs among 20,870 live births were recorded for the years 2000–2004 inclusive in the study region. In each of the years assessed, BD prevalence showed an increasing trend with higher categories of TTHM exposure, with the exception of 2001 (Table 3).

### Risk estimates

Women living in high-TTHM areas at the time of birth of their child showed an increased risk of any BD (adjusted OR = 1.22; 95% CI, 1.01–1.48) compared with women living in low-TTHM areas. For individual BDs, we identified a significantly elevated OR for any cardiovascular BD (adjusted OR = 1.62; 95% CI, 1.04–2.51) for women living in high-TTHM areas compared with women living in low-TTHM areas. Table 4 summarizes all results. We evaluated maternal age as an independent risk factor for BDs and a potential confounder in this analysis. Using maternal age (in 5-year age strata) as a predictor, we calculated an OR of 1.03 (95% CI, 0.98–1.09) for BD overall. Because average maternal age varied slightly by region, we included it as an individual-level covariate in the final model. For the full model with TTHM exposures included, we undertook restricted analysis on mothers < 35 years of age to explore possible impacts of advanced maternal age. ORs for the restricted population were comparable with the original analysis (data not shown). We also conducted mixed-effect multilevel analyses, but risk estimates did not substantially differ from those using the original regression models, with persistence of the significant findings for overall BD and cardiovascular BD (data not shown).

## Discussion

The results from this record-based prevalence study indicate that women living in high-TTHM areas in Perth at the time of birth of their baby have a 22% greater risk of having a baby with any BD. More specifically, classification in the high-exposure group is associated with an increased risk of 62% for having a baby with a cardiovascular defect. In this analysis, results for musculoskeletal and urogenital defects are suggestive but non-significant, with adjusted ORs of 1.48 (95% CI, 0.99–2.21) and 1.40 (95% CI, 0.98–1.99), respectively.

These findings suggest that there may indeed be an area-related effect in Perth, attributable to the city’s heterogeneous water supply and quality. As discussed, the differential exposure patterns most likely relate to the quantity of precursors in the source waters, which display a rough latitudinal gradient. The analyses of BD rates by year also suggest that, except for 2001, the trend toward high defect rates for higher THM levels has been consistent. By international standards, a high proportion of the THMs in the areas tested were in the brominated form. Brominated THMs are thought to possess a greater heath risk compared with chloroform, and this largely relates to differences in their metabolism and toxicokinetics (Bull et al. 1995). Reproductive and developmental toxicity has been observed in particular with BDCM (Bielmeier et al. 2001, 2003; Chen et al. 2004; Hunter 2002; Narotsky et al. 1997). We assessed low, medium, and high water consumption in a subpopulation of pregnant women living in Perth in 2004–2005 (data not shown). Based on the ingestion rates for the surveyed population, an exposure model estimated that residence in the highest TTHM exposure group ingesting the highest self-reported intake amount of 4.67 L/day will expose a pregnant women to an equivalent of 3.85 μg TTHM/day. Using this estimation technique, an estimated 3.47 μg TTHM/day (90.1%) are brominated THMs (Chisholm K, Cook A, Weinstein P, unpublished data).

These findings are compatible with a critical review that found evidence for a weak association between the presence of any BD and TTHMs; the risk appeared highest for urinary tract BDs specifically (Graves et al. 2001). A review published in 2006 formed similar conclusions (Tardiff et al. 2006). Limited research has been conducted on musculoskeletal defects, with weak associatons reported (Graves et al. 2001; Tardiff et al. 2006). The present study showed no significance for nervous system defects, which contrasts with results from Dodds and King (2001) that neural tube defects were significantly associated with exposure to > 20 μg/L BDCM (OR = 2.5; 95% CI, 1.2–5.1), the same level of BDCM we found in Perth water supplies (Table 2).

Recent literature suggests that lower socioeconomic indices are often predictive of higher BD rates (Carmichael et al. 2003; Wasserman et al. 1998), but in this study our regression models did not suggest an obvious relationship between SEIFA, a composite measure of socioeconomic deprivation available at census district levels (which contain, on average, 225 households), and rates of BDs in the suburbs examined. In this study, we aggregated the SEIFA codes to calculate an average by post code, which was the spatial unit of BD data (Table 2 summarizes the number of post codes per exposure area). In relation to the exposure, areas with higher socioeconomic (SEIFA) scores commonly fell within the highest THM category; that is, mothers resident in the more affluent suburbs were either just as or more likely to be highly exposed to THMs compared with those in socioeconomically deprived suburbs (Table 2). This suggests that socioeconomic factors themselves are not a plausible explanation for the observed findings, and—if anything—the spatial relationship between high socioeconomic status and low quality water in the study area may have tended to attenuate the risk estimate. The particular hospital or clinic used for delivery is also unlikely to be a source of error because the system for collecting BD data is consistent across clinics and routinely audited. In addition, differential maternity/obstetric hospital use by location of residence (and hence exposure) is unlikely given the small number of such hospitals in the catchment areas used for the study. We assessed potential confounding by maternal age, but it was not a significant predictor of BDs in most full exposure models (including those with positive findings for THM exposure). The average age of the mothers varied slightly by TTHM exposure region, but we adjusted for possible confounding effects at the individual level using the deidentified case data.

We acknowledge several limitations in this analysis. Because we used an ecologic exposure measure in this study, the use of residential TTHMs to assign individual exposure raises the possibility of exposure misclassification. For example, women may not have lived in the area at the critical time window relevant to formation of BDs (at conception and first trimester for most BDs). A survey of 78 pregnant women in Perth that we conducted in 2004–2005 (data not included) found that 15.6% of participants reported moving residence during their pregnancy. This finding is similar to recent data reported from a cohort of 584 pregnant women recruited from within Sydney between 2004 and 2006. Of these women, 18% moved residences, although this estimate includes residential movement up to 6 months postpartum (Raynes-Greenow et al. 2007). If exposure misclassification has occurred as a result of migration, it is likely to be nondifferential, so risk estimates presented in this study may be overly conservative. Because we have not accounted for individual exposure assessment, we have not taken into account contact with other disinfected water sources (e.g., through swimming and work-related exposures). However, a small survey of 54 pregnant women in metropolitan Perth we conducted in 2004 (data not shown) indicated that 68.5% consumed tap water during their pregnancy, suggesting that bottled water is not a commonly used alternative among this population. Rainwater is not commonly used in Western Australia. A total of 96.3% of the women interviewed used publicly supplied water for showering and bathing, indicating that contact with the town supply remains relevant given that inhalation and dermal absorption are important routes of exposure to THMs (Meek et al. 2002; Weisel and Chen 1994; Xu et al. 2002; Xu and Weisel 2005).

The measurements used were not taken during the same time period as the health outcome, and limited public availability of complete TTHM levels in Perth precludes the reliable use of historical data. The limited DBP data published for the Perth region is consistent with our exposure estimates, but this historical data set consists of, on average, four samples (or fewer) per year and covers only half of the suburbs that we tested in our study (Water Corporation of Western Australia 2002–2003). Many factors are known to affect DBP formation, including pH, contact time, temperature, concentration and properties of NOM, concentration of both chlorine and residual chlorine, and concentration of bromide, and these may be subject to variation seasonally and from year to year (Krasner et al. 1989; Singer 1993). We observed elevated THM levels during the summer months, and we consistently recorded the highest levels at sites within the high-TTHM regions. Because the increases and decreases in THM levels occurred concurrently across all regions by season, the differences between average levels observed within low, medium, and high regions remained consistent. For confidentiality, birth and BD data were not available by date of birth, so we could not subclassify based on season.

Finally, we extracted BD data for this study in 2007, so for children born after 2001, defects not recognized until later in their lives have yet to be ascertained and included in the registry. However, there is no reason to suppose that this process would be differential by post code, and any incomplete ascertainment for more recent years of birth would be similar for all study locations. Western Australian also has only one centralized Birth Defects Registry, with consistent ascertainment and quality assurance protocols, making substantial variability by region unlikely, particularly within the metropolitan area.

It is reasonable to argue that public health benefits from chlorination in terms of microbiologic safety prevail over the conflicting evidence of potential health risks associated with DBP exposure (Tardiff et al. 2006). However, in Australia and other centers that permit elevated DBP levels, parts of the population are exposed to levels higher than those considered prudent by other industrialized countries. We suggest that national or regional authorities that permit higher TTHM levels may need to reexamine their water quality guidelines to reduce avoidable adverse birth outcomes. If a true elevation in risk is confirmed, a massive and costly engineering “fix” to reduce DBP exposure may not necessarily be warranted. Public health interventions in problem regions of low water quality and availability may be as simple as recommending and/or providing bottled water for pregnant women or ensuring adequate filtration of contaminants before consumption.

## Figures and Tables

**Table 1:** Review of principal associations and risk measures for DBP exposure and BDs.

Study design, region, reference	Exposure range	Defect type	Risk estimate (95% CI)
Retrospective cohort, Canada (Dodds et al. 1999)	High (> 100 μg/L) versus low (< 50 μg/L) THM levels	Neural tube defects	2.96 (1.26–6.62)
		Major cardiac defects	1.83 (0.97–3.92)
		Oral cleft defects	3.17 (1.18–7.26)
Case–control, USA (Klotz and Pyrch 1999)	High (> 40 μg/L) and low (< 5 μg/L) THMs	Neural tube defects	2.10 (1.10–4.00)
	High (> 3 μg/L) and low (< 0.5 μg/L) HANs	Neural tube defects	1.30 (0.60–2.50)
	High (> 35 μg/L) and low (< 3 μg/L) HAAs	Neural tube defects	1.20 (0.50–2.60)
Retrospective cohort, Sweden (Kallen and Robert 2000)	Chlorinated versus nonchlorinated water	Major cardiac defects	1.10 (0.90–1.30)
		Oral cleft defects	1.10 (0.80–1.60)
Retrospective cohort, Canada (Dodds and King 2001)	High (> 20 μg/L) and low (< 5 μg/L) BDCM	Neural tube defects	2.50 (1.20–5.10)
		Major cardiac defects	0.30 (0.20–0.70)
		Oral cleft defects	0.60 (0.20–0.90)
Cross-sectional, Norway (Hwang et al. 2002)	Chlorination and color versus nonchlorinated	All sentinel anomalies	1.18 (1.02–1.36)
		Ventricular septal defects	1.81 (1.05–3.09)
		Respiratory defects	1.89 (1.00–3.58)
		Urinary tract defects	1.46 (1.00–2.13)
Case–control, Sweden (Cedergren et al. 2002)	High (> 10 μg/L ) and low (< 5 μg/L) THMs	Cardiac defects	1.30 (1.08–1.56)
Case–control, USA (Shaw et al. 2003)	50–74 μg/L THMs	Study 1: Neural tube defects	0.62 (0.26–1.50)
		Study 2: Neural tube defects	1.80 (0.26–1.50)
		Oral cleft defects	1.00 (0.32–3.40)
Registry-based cohort, England and Wales (Niewenhuijsen et al. 2008)	High (4 μg/L) versus low (< 2 μg/L) bromoform	Major cardiac defects	1.18 (1.00–1.39)

**Table 2:** Demographics and TTHM concentrations for exposure areas.

	Area of TTHM exposure		
Characteristic	Low	Medium	High
No. of post codes included in exposure area	9	5	11
Average maternal age range (years)	25–29	30–34	30–34
SEIFA^a^			
Range	945–1,026	1,002–1,100	873–1,082
Average	994.2	1033.6	982.1
Median	989.8	1028.0	997.6
Average THM concentration (μg/L)			
TTHM	54.0	109.3	137.0
Chloroform	2.0	9.0	15.0
Bromoform	31.0	39.5	39.0
BDCM	5.0	19.5	29.0
DBCM	17.0	44.0	54.5
Median THM concentration (μg/L)			
TTHM	51.0	115.0	140.0
Chloroform	1.0	8.2	11.0
Bromoform	29.0	38.0	40.0
BDCM	3.3	19.0	27.0
DBCM	16.0	44.5	53.0
Maximum individual site THM concentration (μg/L)			
TTHM	140.0	160.0	190.0
Chloroform	5.2	19.0	52.0
Bromoform	54.0	66.0	78.0
BDCM	13.0	44.0	57.0
DBCM	28.0	72.0	81.0
Minimum individual site THM concentration (μg/L)			
TTHM	36.0	39.0	61.0
Chloroform	1.0	1.0	2.8
Bromoform	20.0	6.3	9.6
BDCM	2.6	3.1	6.8
DBCM	10.0	12.0	18.0
25th percentile THM concentration (μg/L)			
TTHM	42.0	93.25	120.0
Chloroform	1.0	6.20	8.1
Bromoform	24.0	33.50	20.0
BDCM	3.2	14.75	19.0
DBCM	13.0	34.75	46.0
75th percentile THM concentration (μg/L)			
TTHM	62.0	130.0	150.0
Chloroform	3.15	10.25	16.0
Bromoform	34.0	47.25	53.0
BDCM	3.8	24.00	35.0
DBCM	18.0	53.25	62.0

a Low values indicate areas of disadvantage, and high values indicate areas of advantage.

**Table 3:** Number of births, BDs, and BD prevalence for TTHM exposure areas for births within the Perth metropolitan area, 2000–2004 inclusive.

		Year					
TTHM exposure group	Births and BDs	2000	2001	2002	2003	2004	2000–2004
Low (< 60 μg/L)	TB (*n*)	518	556	572	639	659	2,944
	BD (*n*)	31	31	19	24	29	134
	BDP (%)	6.4	5.6	3.3	3.8	4.4	4.6
Medium (> 60 to < 130 μg/L)	TB (*n*)	958	934	930	945	981	4,748
	BD (*n*)	58	60	35	42	40	235
	BDP (%)	6.4	6.4	3.8	4.4	4.1	5.0
High (≥ 130 μg/L)	TB (*n*)	2,638	2,666	2,593	2,557	2,724	13,178
	BD (*n*)	174	149	146	127	132	728
	BDP (%)	7.1	5.6	5.6	5.0	4.8	5.5

Abbreviations: BDP, BD prevalence for exposure area; TB, total number of births for exposure area.

**Table 4:** ORs (95% CIs) for the association between TTHM exposure and any BD or individual BD, 2000–2004 inclusive.

Birth outcome^a^	Case number^b^	Adjusted OR (95% CI)^c^
Any BD		
Low	134	1.00
Medium	235	0.98 (0.75–1.28)
High	728	1.22 (1.01–1.48)*
Cardiovascular (BPA 74500–74799)		
Low	24	1.00
Medium	55	1.00 (0.55–1.81)
High	181	1.62 (1.04–2.51)*
Musculoskeletal (BPA 75400–75699)		
Low	29	1.00
Medium	53	1.05 (0.60–1.83)
High	200	1.48 (0.99–1.21)
Gastrointestinal (BPA 74900–75199)		
Low	11	1.00
Medium	24	1.27 (0.55–2.96)
High	66	1.20 (0.63–2.30)
Urogenital (BPA 75200–75399)		
Low	40	1.00
Medium	76	1.09 (0.68–1.77)
High	235	1.40 (0.98–1.99)
Nervous system (BPA 74000–74299)		
Low	6	1.00
Medium	15	1.78 (0.55–5.80)
High	38	1.08 (0.41–2.85)
Respiratory system (BPA 74800–74899)		
Low	2	1.00
Medium	3	1.06 (0.13–8.87)
High	12	0.88 (0.18–4.18)
Integument congenital anomalies (BPA 75700–75799)		
Low	13	1.00
Medium	15	0.91 (0.36–2.33)
High	8	0.95 (0.49–1.83)

a Low, < 60 μg/L; medium, > 60 to < 130 μg/L; high, ≥ 130 μg/L.

b Total births for each exposure area: low, 2,944; medium, 4,748; high, 13,178.

c Adjusted for maternal age and socioeconomic status.

* *p* < 0.05.

## References

[b1-ehp-116-1267] Agency for Toxic Substances and Disease Registry (1993). Toxicological Profile for Chloroform.

[b2-ehp-116-1267] Australian Bureau of Statistics (2001). Socio-Economic Indexes for Areas 2001 (SEIFA 2001).

[b3-ehp-116-1267] Bielmeier SR, Best DS, Guidici DL, Narotsky MG (2001). Pregnancy loss in the rat caused by bromodichloromethane. Toxicol Sci.

[b4-ehp-116-1267] Bielmeier SR, Murr AS, Best DS, Goldman JM, Narotsky MG (2003). Effects of bromodichloromethane (BDCM) on *ex vivo* luteal function in the pregnant F344 rat. Toxicologist.

[b5-ehp-116-1267] Bove F, Shim Y, Zeitz P (2002). Drinking water contaminants and adverse pregnancy outcomes: a review. Environ Health Perspect.

[b6-ehp-116-1267] Bower C, Rudy E, Callaghan A, Cosgrove P, Quick J (2007). Report of the Birth Defects Registry of Western Australia 1980–2006. No. 14.

[b7-ehp-116-1267] Bower C, Ryan A, Rudy E (2001). Ascertainment of pregnancies terminated because of birth defects: effect on completeness of adding a new source of data. Teratology.

[b8-ehp-116-1267] Bower C, Silva D, Henderson TR, Ryan A, Rudy E (2000). Ascertainment of birth defects: the effect on completeness of adding a new source of data. J Paediatr Child Health.

[b9-ehp-116-1267] British Paediatric Association (1979). British Paediatric Association Classification of Diseases.

[b10-ehp-116-1267] Bull RJ, Birnbaum LS, Cantor KP, Rose JB, Butterworth BE, Pegram R (1995). Water chlorination: essential process or cancer hazard?. Fundam Appl Toxicol.

[b11-ehp-116-1267] Butterworth BE (2005). Science-based risk assessments for drinking water disinfection by-products. Environ Res.

[b12-ehp-116-1267] Butterworth BE, Bogdanffy MS (1999). A comprehensive approach for integration of toxicity and cancer risk assessments. Regul Toxicol Pharmacol.

[b13-ehp-116-1267] Carmichael SL, Nelson V, Shaw GM, Wasserman CR, Croen LA (2003). Socio-economic status and risk of conotruncal heart defects and orofacial clefts. Paediatr Perinat Epidemiol.

[b14-ehp-116-1267] Cedergren MI, Selbing AJ, Lofman O, Kallen BA (2002). Chlorination byproducts and nitrate in drinking water and risk for congenital cardiac defects. Environ Res.

[b15-ehp-116-1267] Chen J, Thirkill TL, Lohstroh PN, Bielmeier SR, Narotsky MG, Best DS (2004). Bromodichloromethane inhibits human placental trophoblast differentiation. Toxicol Sci.

[b16-ehp-116-1267] Chen WJ, Weisel C (1998). Halogenated DBP concentrations in a distribution system. J Am Water Works Assoc.

[b17-ehp-116-1267] Da Silva ML, Charest-Tardif G, Krishnan K, Tardif R (2000). Evaluation of the pharmacokinetic interactions between orally administered trihalomethanes in the rat. J Toxicol Environ Health A.

[b18-ehp-116-1267] DeAngelo AB, Geter DR, Rosenberg DW, Crary CK, George MH (2002). The induction of aberrant crypt foci (ACF) in the colons of rats by trihalomethanes administered in the drinking water. Cancer Lett.

[b19-ehp-116-1267] Dodds L, King WD (2001). Relation between trihalomethane compounds and birth defects. Occup Environ Med.

[b20-ehp-116-1267] Dodds L, King W, Allen AC, Armson BA, Fell DB, Nimrod C (2004). Trihalomethanes in public water supplies and risk of stillbirth. Epidemiology.

[b21-ehp-116-1267] Dodds L, King W, Woolcott C, Pole J (1999). Trihalomethanes in public water supplies and adverse birth outcomes. Epidemiology.

[b22-ehp-116-1267] Geter DR, Chang LW, Hanley NM, Ross MK, Pegram RA, DeAngelo AB (2004). Analysis of in vivo and in vitro DNA strand breaks from trihalomethane exposure. J Carcinog.

[b23-ehp-116-1267] Goebell PJ, Villanueva CM, Rettenmeier AW, Rubben H, Kogevinas M (2004). Environmental exposure, chlorinated drinking water, and bladder cancer. World J Urol.

[b24-ehp-116-1267] Graves CG, Matanoski GM, Tardiff RG (2001). Weight of evidence for an association between adverse reproductive and developmental effects and exposure to disinfection by-products: a critical review. Regul Toxicol Pharmacol.

[b25-ehp-116-1267] Heitz A, Joll C, Alexander R, Kagi R, Swift R, Spark K (2001). Characterisation of aquatic natural organic matter in some Western Australian drinking water sources. Understanding and Managing Organic Matter in Soils, Sediments and Waters.

[b26-ehp-116-1267] Holman CD, Bass AJ, Rouse IL, Hobbs MS (1999). Population-based linkage of health records in Western Australia: development of a health services research linked database. Aust N Z J Public Health.

[b27-ehp-116-1267] Hunter ES (2002). Developmental consequences of exposure to disinfection by-products in animal models. Toxicologist.

[b28-ehp-116-1267] Hwang BF, Jaakkola JJ (2003). Water chlorination and birth defects: a systematic review and meta-analysis. Arch Environ Health.

[b29-ehp-116-1267] Hwang BF, Magnus P, Jaakkola JJ (2002). Risk of specific birth defects in relation to chlorination and the amount of natural organic matter in the water supply. Am J Epidemiol.

[b30-ehp-116-1267] International Life Sciences Institute (1995). Report of Epidemiological Workshop for Disinfection By-products and Reproductive Effects.

[b31-ehp-116-1267] Kallen BA, Robert E (2000). Drinking water chlorination and delivery outcome—a registry-based study in Sweden. Reprod Toxicol.

[b32-ehp-116-1267] Keegan T, Whitaker H, Nieuwenhuijsen MJ, Toledano MB, Elliott P, Fawell J (2001). Use of routinely collected data on trihalomethane in drinking water for epidemiological purposes. Occup Environ Med.

[b33-ehp-116-1267] Keegan TE, Simmons JE, Pegram RA (1998). NOAEL and LOAEL determinations of acute hepatotoxicity for chloroform and bromodichloromethane delivered in an aqueous vehicle to F344 rats. J Toxicol Environ Health A.

[b34-ehp-116-1267] King WD, Dodds L, Allen AC (2000). Relation between stillbirth and specific chlorination by-products in public water supplies. Environ Health Perspect.

[b35-ehp-116-1267] King WD, Dodds L, Allen AC, Armson BA, Fell D, Nimrod C (2005). Haloacetic acids in drinking water and risk for stillbirth. Occup Environ Med.

[b36-ehp-116-1267] Klotz JB, Pyrch LA (1999). Neural tube defects and drinking water disinfection by-products. Epidemiology.

[b37-ehp-116-1267] Krasner SW, McGuire MJ, Jacaugelo JG, Patania NL, Reagen KM, Aieta EM (1989). The occurrence of disinfection byproducts in US drinking water. J Am Water Works Assoc.

[b38-ehp-116-1267] Landi S, Hanley NM, Warren SH, Pegram RA, DeMarini DM (1999). Induction of genetic damage in human lymphocytes and mutations in Salmonella by trihalomethanes: role of red blood cells and GSTT1-1 polymorphism. Mutagenesis.

[b39-ehp-116-1267] Magnus P, Jaakkola JJ, Skrondal A, Alexander J, Becher G, Krogh T (1999). Water chlorination and birth defects. Epidemiology.

[b40-ehp-116-1267] Meek ME, Beauchamp R, Long G, Moir D, Turner L, Walker M (2002). Chloroform: exposure estimation, hazard characterization, and exposure-response analysis. J Toxicol Environ Health B Crit Rev.

[b41-ehp-116-1267] Narotsky MG, Pegram RA, Kavlock RJ (1997). Effect of dosing vehicle on the developmental toxicity of bromodichloromethane and carbon tetrachloride in rats. Fundam Appl Toxicol.

[b42-ehp-116-1267] NHMRC (2004). National Water Quality Management Strategy Australian Drinking Water Guidelines.

[b43-ehp-116-1267] Nieuwenhuijsen MJ, Northstone K, Golding J (2002). Swimming and birth weight. Epidemiology.

[b44-ehp-116-1267] Nieuwenhuijsen MJ, Toledano MB, Bennett J, Best N, Hambly P, de Hoogh C (2008). Chlorination disinfection byproducts and risk of congenital anomalies in England and Wales. Environ Health Perspect.

[b45-ehp-116-1267] Nieuwenhuijsen MJ, Toledano MB, Eaton NE, Fawell J, Elliott P (2000). Chlorination disinfection byproducts in water and their association with adverse reproductive outcomes: a review. Occup Environ Med.

[b46-ehp-116-1267] Piccolo A, Spiteller M (2003). Electrospray ionization mass spectrometry of terrestrial humic substances and their size fractions. Anal Bioanal Chem.

[b47-ehp-116-1267] Raynes-Greenow CH, Nassar N, Roberts CL, Levett K (2007). Do mothers move? A problem for follow-up. J Paediatr Child Health.

[b48-ehp-116-1267] Reif JS, Hatch MC, Bracken M, Holmes LB, Schwetz BA, Singer PC (1996). Reproductive and developmental effects of disinfection by-products in drinking water. Environ Health Perspect.

[b49-ehp-116-1267] Rodriguez MJ, Serodes JB (2001). Spatial and temporal evolution of trihalomethanes in three water distribution systems. Water Res.

[b50-ehp-116-1267] Rodriguez MJ, Serodes JB, Levallois P (2004). Behavior of trihalomethanes and haloacetic acids in a drinking water distribution system. Water Res.

[b51-ehp-116-1267] Rook JJ (1974). Formation of haloforms during chlorination of natural waters. Water Treat Exam.

[b52-ehp-116-1267] Shaw GM, Ranatunga D, Quach T, Neri E, Correa A, Neutra RR (2003). Trihalomethane exposures from municipal water supplies and selected congenital malformations. Epidemiology.

[b53-ehp-116-1267] Singer PC, Cruan GF (1993). Formation and characterization of disinfection byproducts. Safety of Water Disinfection: Balancing Microbial Risks.

[b54-ehp-116-1267] Tardiff RG, Carson ML, Ginevan ME (2006). Updated weight of evidence for an association between adverse reproductive and developmental effects and exposure to disinfection by-products. Regul Toxicol Pharmacol.

[b55-ehp-116-1267] Thurman E, Malcolm R (1981). Preparative isolation of aquatic humic substances. Environ Sci Technol.

[b56-ehp-116-1267] U.S. EPA (2003). Method 5030C: Purge-and-Trap for Aqueous Samples.

[b57-ehp-116-1267] U.S. EPA (2004). 2004 Edition of the Drinking Water Standards and Health Advisories.

[b58-ehp-116-1267] Villanueva CM, Fernandez F, Malats N, Grimalt JO, Kogevinas M (2003). Meta-analysis of studies on individual consumption of chlorinated drinking water and bladder cancer. J Epidemiol Community Health.

[b59-ehp-116-1267] Waller K, Swan SH, DeLorenze G, Hopkins B (1998). Trihalomethanes in drinking water and spontaneous abortion. Epidemiology.

[b60-ehp-116-1267] Wasserman CR, Shaw GM, Selvin S, Gould JB, Syme SL (1998). Socioeconomic status, neighborhood social conditions, and neural tube defects. Am J Public Health.

[b61-ehp-116-1267] Water Corporation of Western Australia (2002–2003). Water Corporation Drinking Water Quality Annual Report.

[b62-ehp-116-1267] Weisel CP, Chen WJ (1994). Exposure to chlorination by-products from hot water uses. Risk Anal.

[b63-ehp-116-1267] Weisel CP, Jo WK (1996). Ingestion, inhalation, and dermal exposures to chloroform and trichloroethene from tap water. Environ Health Perspect.

[b64-ehp-116-1267] Wong S, Hanna JV, King S, Carroll TJ, Eldridge RJ, Dixon DR (2002). Fractionation of natural organic matter in drinking water and characterization by ^13^C cross-polarization magic-angle spinning NMR spectroscopy and size exclusion chromatography. Environ Sci Technol.

[b65-ehp-116-1267] Xu X, Mariano TM, Laskin JD, Weisel CP (2002). Percutaneous absorption of trihalomethanes, haloacetic acids, and haloketones. Toxicol Appl Pharmacol.

[b66-ehp-116-1267] Xu X, Weisel CP (2005). Human respiratory uptake of chloroform and haloketones during showering. J Expo Anal Environ Epidemiol.

[b67-ehp-116-1267] Yang CY, Cheng BH, Tsai SS, Wu TN, Lin MC, Lin KC (2000). Association between chlorination of drinking water and adverse pregnancy outcome in Taiwan. Environ Health Perspect.

